# Impact of temporal probability in 4D dose calculation for lung tumors

**DOI:** 10.1120/jacmp.v16i6.5517

**Published:** 2015-11-08

**Authors:** Ouided Rouabhi, Mingyu Ma, John Bayouth, Junyi Xia

**Affiliations:** ^1^ Department of Biomedical Engineering University of Iowa Iowa City IA; ^2^ Department of Physics University of Iowa Iowa City IA; ^3^ Department of Human Oncology University of Wisconsin – Madison Madison WI; ^4^ Department of Radiation Oncology University of Iowa Iowa City IA USA

**Keywords:** 4D CT, dose calculation, SBRT, 4D dose, deformable image registration

## Abstract

The purpose of this study was to evaluate the dosimetric uncertainty in 4D dose calculation using three temporal probability distributions: uniform distribution, sinusoidal distribution, and patient‐specific distribution derived from the patient respiratory trace. Temporal probability, defined as the fraction of time a patient spends in each respiratory amplitude, was evaluated in nine lung cancer patients. Four‐dimensional computed tomography (4D CT), along with deformable image registration, was used to compute 4D dose incorporating the patient's respiratory motion. First, the dose of each of 10 phase CTs was computed using the same planning parameters as those used in 3D treatment planning based on the breath‐hold CT. Next, deformable image registration was used to deform the dose of each phase CT to the breath‐hold CT using the deformation map between the phase CT and the breath‐hold CT. Finally, the 4D dose was computed by summing the deformed phase doses using their corresponding temporal probabilities. In this study, 4D dose calculated from the patient‐specific temporal probability distribution was used as the ground truth. The dosimetric evaluation matrix included: 1) 3D gamma analysis, 2) mean tumor dose (MTD), 3) mean lung dose (MLD), and 4) lung V20. For seven out of nine patients, both uniform and sinusoidal temporal probability dose distributions were found to have an average gamma passing rate >95% for both the lung and PTV regions. Compared with 4D dose calculated using the patient respiratory trace, doses using uniform and sinusoidal distribution showed a percentage difference on average of −0.1%±0.6% and −0.2%±0.4% in MTD, −0.2%±1.9% and −0.2%±1.3% in MLD, 0.09%±2.8% and −0.07%±1.8% in lung V20, −0.1%±2.0% and 0.08%±1.34% in lung V10, 0.47%±1.8% and 0.19%±1.3% in lung V5, respectively. We concluded that four‐dimensional dose computed using either a uniform or sinusoidal temporal probability distribution can approximate four‐dimensional dose computed using the patient‐specific respiratory trace.

PACS number: 87.55.D‐

## I. INTRODUCTION

The American Cancer Society^(1)^ estimates that more than 221,000 new cases of lung and bronchus cancers will be diagnosed in 2015. For many of these patients, treatment will involve radiation therapy, such as intensity‐modulated radiation therapy (IMRT) and stereotactic body radiation therapy (SBRT). These techniques aim to deliver a high radiation dose to the tumor, while minimizing the radiation dose to healthy tissues.^2^, ^3^, ^4^, ^5^ One obstacle, however, remains to be the uncertainty in tumor position introduced from the patient's respiratory motion. Lung cancers are known to be most affected by respiration‐induced motion.^(6)^ Studies^7^, ^8^, ^9^, ^10^, ^11^ have shown that lung tumors can move more than 10 mm during respiration, with the greatest tumor motion occurring in the lower lobes of the lung. Studies^8^, ^11^, ^12^ have also shown that the percentage of time spent in each respiratory amplitude is not uniform; patients spend more time in the exhalation phase than in the inhalation phase. In order to ensure correct dose coverage during treatment delivery, the extent of the tumor motion within breathing cycles must be evaluated.^(13)^

Conventionally, a free‐breathing computed tomography (CT) scan has been used for treatment planning.^14^, ^15^ However, tumor motion can introduce severe artifacts, resulting in distortion of the tumor and inaccurate assessment of the tumor's location.^16^, ^17^ By acquiring breath‐hold CT scans, motion artifacts may be reduced; however, uncertainty in tumor position still remains.^(14)^ In order to compensate for respiration‐induced uncertainties in dose distribution, additional treatment margins are often added to the target volume, resulting in an increased dose to the healthy tissue.^(15)^

With the introduction of four‐dimensional computed tomography (4D CT), also known as multiphase CT scanning, treatment centers are now able to better account for respiratory motion. During 4D CT imaging, both the CT projections and the patient's respiratory trace are acquired. The respiratory trace is then used to associate each CT projection with either the respiratory amplitude (amplitude binning) or the respiratory phase (phase binning). A binned CT image, also called a phase CT, is then reconstructed for each respiratory amplitude, in the case of amplitude binning, or time point, in the case of phase binning.^18^, ^19^, ^20^ To calculate the 4D treatment plan, dose is firstly computed for each phase CT, then mapped to the breath‐hold CT by deformable image registration. The 4D dose can then be calculated by pointwise summation of the phase dose distributions using respective weighting for each phase. In cases where phase binning is used for sorting the 4D CT projections, each phase corresponds to the same time interval; therefore phase doses should be weighted equally. However, in cases where amplitude binning is used for sorting, each phase dose should be weighted with respect to the fraction of time the patient spends at that particular respiratory amplitude (i.e., the temporal probability).^(13)^ Since it has already been shown that patients do not spend an equal amount of time in each respiratory phase,^8^, ^11^, ^12^ ideally a patient‐specific temporal probability distribution should be derived from the patient's respiratory trace. However, in cases where the patient's respiratory trace is not readily available, it is unclear whether an approximated temporal probability distribution may be used, or how sensitive the 4D dose calculation is to the underlying temporal probability distribution.

Several methods^(15)^ have been proposed for dose calculations that consider respiration‐introduced intrafractional tumor motion. Each of these features a technique for incorporating tumor motion, either by deriving patient‐specific temporal probabilities from the respiratory trace^(15)^ or by modeling the tumor's trajectory. Many previous studies incorporating motion have assumed a sinusoidal respiratory model.^21^, ^22^, ^23^ Lujan et al.,^(22)^ George et al.,^(21)^ and Bortfeld et al.^(23)^ presented sinusoidal probability distribution functions (PDFs), which modeled the tumor's motion as being periodic, while asymmetric, accounting for increased time spent in the exhalation phase. These approaches featured a convolution of the static dose distribution with the PDF of the tumor's motion.^15^, ^21^, ^22^, ^23^ When comparing different treatment planning algorithms, Lax et al.^(24)^ used the convolution method to compare dose distributions for SBRT cases using four different probability distribution functions: linear, harmonic oscillator, patient data with fixed amplitude and frequency, and patient data with variances in amplitude and motion pattern. They found that the differences in dose distributions were relatively small among the four PDFs. However, this study was limited to phantom images. More recent 4D dose calculation studies have employed 4D CT images. Rietzel et al.^(14)^ and Guckenberger et al.^(13)^ used deformable image registration techniques to calculate delivered dose in the presence of respiratory motion. They assumed equal weighting between respiratory phases because the binned CT data were uniformly distributed across a respiratory cycle. Similarly, Guerrero et al.^(25)^ also used equal fractional weighting when combining doses for 4D mapping in their phantom thoracic radiotherapy study, and Flampouri et al.^(20)^ used similar 4D calculation methods to study delivered dose in IMRT patients. To the best of our knowledge, no studies have yet been conducted to explicitly evaluate the dosimetric effects of different temporal probabilities using 4D CT. In this study, we investigated the dosimetric uncertainty in 4D dose calculation using three different temporal probability distributions: uniform distribution, sinusoidal distribution, and patient‐specific distribution derived from the patient respiratory trace.

## II. MATERIALS AND METHODS

Following institutional review board approval, treatment plans for nine lung cancer patients were retrospectively evaluated. A summary of patient characteristics is listed in Table 1, where maximum tumor motion varied from 3 to 23 mm. Among the nine patients, five were treated using stereotactic body radiation therapy, while the remaining four were treated using intensity‐modulated radiation therapy. Gated radiation therapy was used in four of the nine patients.

Patient CT images were acquired using the Siemens Biograph PET‐CT scanner (Siemens Medical System, Knoxville, TN). For each patient, a breath‐hold CT scan at the end of exhale was first taken, followed by a 4D CT scan, during which the patient's respiratory trace was recorded using a commercially available strain gauge pressure sensing system (Anzai Medical Co. Ltd, Tokyo, Japan) attached to the upper abdominal region using an elastic belt. Retrospective sorting of the 4D CT projections was performed using the CT console. Amplitude‐based binning was used for image reconstruction. For each 4D CT scan, 10 phase CT images were reconstructed, representing 10 different respiratory amplitudes.

Table 2 lists the planning methods and prescriptions for the SBRT patients. The prescription dose varied from 8 Gy/fraction to 18 Gy/fraction with treatment fractions varied from 3 to 5 fractions. Both static beams and IMRT were used in the SBRT planning.

**Table 1:** Summary of patient characteristics

*Patient*	*Lung Volume (cm^3^)*	*PTV Volume (cm^3^)*	*Tumor Motion (mm)*	*Treatment*	*Gated Therapy*
P1	816	14	6	SBRT	Yes
P2	2,103	236	15	IMRT	Yes
P3(LT)	1,872	160	23	SBRT	Yes
P3(RT)	2,383	17	3	SBRT	No
P4	791	97	10	IMRT	No
P5	733	578	3	IMRT	No
P6	1,859	110	8	SBRT	No
P7	1,502	33	10	SBRT	No
P8	1,018	115	12	IMRT	Yes
P9	1,695	141	10	SBRT	No

**Table 2:** Summary of planning methods and prescriptions for SBRT patients

*Patient*	*Prescription*	*Static Beam/IMRT*	*Couch Angles*	*# of Beams*
P1	10 Gy×5	Static	0	6
P3(LT)	18 Gy×3	IMRT	0	7
P3(RT)	18 Gy×3	IMRT	0	7
P6	10 Gy×4	IMRT	0	11
P7	18 Gy×3	Static	0, 15, 345	16
P9	8 Gy×5	Static	0, 15, 345	16

### A. 3D dose calculation

The Pinnacle^3^ treatment planning system (Philips Radiation Oncology Systems, Milpitas, CA) was used for treatment planning. The exhale breath‐hold CT scan was used to generate the clinical target volume (CTV). Tumor motion from the 4D CT image was used to define the internal target volume (ITV). Planning target volume (PTV) was delineated by adding 5 mm margins to the anterior–posterior, medial–lateral, and craniocaudal directions of the ITV.

### B. 4D dose calculation

**Figure 1:**
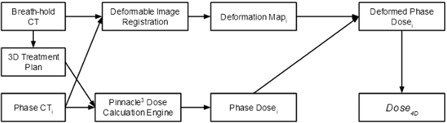
Workflow for 4D dose calculation. First, the dose for each of 10 phase CTs was computed. Next, deformable image registration was used to deform the dose of each phase CT to the breath‐hold CT using the deformation map between the phase CT and the breath‐hold CT. Finally, a 4D dose distribution was computed by summing the deformed phase doses using their corresponding temporal probabilities.

For each patient, three 4D dose distributions were computed, corresponding to three temporal probability distributions. A summary of the workflow is shown in Fig. 1. First, the corresponding dose for each of the 10 binned phase CTs was calculated using the same planning parameters as those used in 3D dose calculation. Next, the 10 binned CTs and their corresponding doses were imported into the VelocityAI software (Velocity Medical Systems, Atlanta, GA). Deformable image registration was then used to compute the deformation map between each binned CT and the breath‐hold CT. This deformation map was applied to the corresponding dose on each binned CT to generate the deformed phase dose. Finally, a MATLAB (MathWorks, Natick, MA) algorithm was used to generate a four‐dimensional dose distribution by performing a pointwise summation of the deformed phase doses with respect to each of three temporal probability distributions: 1) uniform distribution, 2) sinusoidal distribution, and 3) patient‐specific distribution derived from the patient's respiratory trace. The 4D dose can be calculated by

(1) $$Dose_{4D}=\sum_{i}P_{i}\times DeformedPhaseDose_{i}$$

where *i* represents the respiratory phase, $P_{i}$ is the temporal probability for phase i, and $DeformedPhaseDose_{i}$ is the deformed dose for phase i. Note that for patients treated with respiratory gating, only phases within the gating window were included in dose summation.

#### B.1 Temporal probability distribution

Respiratory traces were acquired using a commercially available strain gauge pressure sensing system (Anzai Medical Co. Ltd) fixed to the upper abdomen using an elastic belt. To quantify the patient‐specific temporal probability for each respiratory phase, a MATLAB algorithm was developed to calculate the fraction of time spent within each respiratory amplitude versus the total time of the respiratory trace. For the sinusoidal distribution, a basic sine wave scaled from 0 to 100 (y=50sin⁡(x)+50) was used to model the respiratory trace, and the same function was used to calculate the fraction of time spent within each respiratory amplitude versus the total time of the respiratory trace. For the uniform distribution, temporal probability was defined to be 0.1 for all 10 respiratory amplitudes.

### C. Dosimetric evaluation matrix

In this study, 4D dose calculated using patient‐specific temporal probabilities was used as the ground truth and compared against 4D doses calculated using uniform and sinusoidal temporal probability distributions.

#### C.1 Gamma analysis

3D gamma analysis^26^, ^27^ was used evaluate the 4D dose distributions. Gamma passing rate was defined as the percentage of the volume whose gamma value is equal to or less than 1. In this study, dose difference and distance to agreement criteria were 2% and 2 mm, respectively. An open source MATLAB algorithm (Threaded 3D Gamma, University of Western Ontario, London, ON) was used to calculate a 3D gamma volume, which was then imported into the VelocityAI software for gamma passing rate analysis. 3D gamma analysis was conducted twice for each patient, first comparing the ground truth calculation to dose calculation using a uniform temporal probability distribution and again comparing the ground truth calculation to dose calculation using a sinusoidal temporal probability distribution. In each case, the gamma passing rate was computed for two common structures: the lung and the PTV.

#### C.2 Mean lung dose, mean tumor dose, and lung V20, V10, and V5

As a measure of clinical impact, mean lung dose (MLD), mean tumor dose (MTD), and lung V20, V10, and V5 (percent volume of the lung receiving at least a given dose) were evaluated for each dose distribution. To do so, the VelocityAI software was used to generate and analyze the dose‐volume histogram. Percentage and absolute differences in measures were computed between the ground truth and the approximated dose distributions.

## III. RESULTS

### A. Gamma analysis

**Figure 2:**
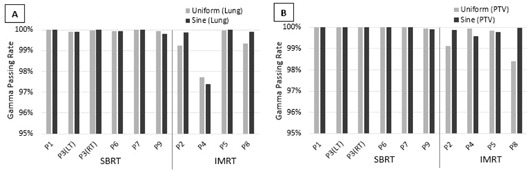
Gamma passing rate using 2% dose difference and 2 mm distance to agreement criteria. For each patient, dose distributions computed using uniform and sinusoidal temporal probability distributions were evaluated against dose distributions computed using the patient's respiratory trace distribution: (a) gamma passing rate for lung; (b) gamma passing rate for PTV.

Figure 2 shows the gamma passing rates. Four‐dimensional dose computed using uniform and sinusoidal temporal probability distributions both passed the 95% gamma acceptance criteria in seven of the nine patients. Gamma passing rates for the lung were greater than 97% for all calculations, with the exception of P4 which had a passing rate greater than 93% when using uniform or sinusoidal distribution. Gamma passing rates were greater than 95% for the PTV, with the exception of the sinusoidal distribution of P4 and the uniform dose distribution of P8 which had gamma passing rates greater than 93% and 92%, respectively.

### B. Mean tumor dose (MTD)

**Figure 3:**
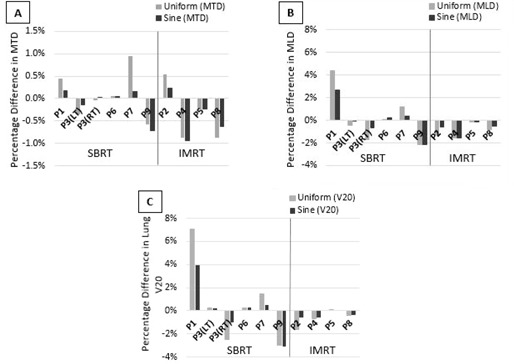
Percentage difference in mean tumor dose (a), mean lung dose (b), and lung V20 (c) for uniform and sinusoidal temporal probability distributions as compared to patient‐specific distributions.

As shown in Fig. 3(a), when compared with calculations using patient‐specific temporal probabilities, those using uniform distribution or sinusoidal distribution showed a percentage difference on average of −0.1%±0.6% and −0.2%±0.4% in MTD, respectively. For all patients, the percentage difference in MTD was less than 1% when using uniform or sinusoidal distribution, and absolute difference in MTD was less than 1 Gy.

### C. Mean lung dose (MLD)

As shown in Fig. 3(b), on average, percentage difference in MLD was found to be −0.2%±1.9% and −0.2%±1.3% when using uniform or sinusoidal dose distribution, respectively. Some patients appeared to exhibit a greater percentage difference in MLD; however, the absolute difference in the calculated measurements for all patients was very small. Patient P1 showed the greatest percentage difference in MLD, 4.4% and 2.7%, respectively, when using uniform and sinusoidal distributions. However, the absolute difference for this patient was only 0.2 Gy and 0.1 Gy, respectively, for uniform and sinusoidal distributions.

### D. Lung V20, V10, and V5

Figure 3(c) shows the percentage difference in patient lung V20 when using uniform and sinusoidal distributions. On average, percentage difference in lung V20 was 0.09%±2.8% and −0.07%±1.8%, respectively, when using uniform or sinusoidal distribution. Patient P1 again showed the greatest percentage difference, 7.1% and 4.0% difference in lung V20, respectively, for uniform and sinusoidal distributions. Again, the absolute difference in value for this patient is very small, 0.5% and 0.28%, respectively, for uniform and sinusoidal distributions. Lung V10 and V5 reflected only slightly greater differences. The average percentage differences in lung V10 and V5 for uniform and sinusoidal distributions were −0.11%±2.0% and −0.08%±1.3% for lung V10, and −0.47%±1.8% and −0.19%±1.3% for lung V5, respectively.

## IV. DISCUSSION

**Figure 4:**
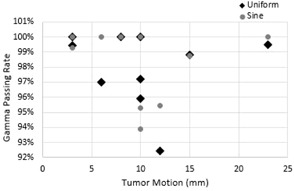
Gamma passing rate of PTV with respect to the magnitude of the tumor motion. Because the majority of gamma passing rates were very similar, no definitive correlations could be drawn between the tumor motion and the gamma passing rate.

Average gamma passing rate for 4D dose calculations computed using both uniform and sinusoidal temporal probability distributions exceeded the 95% gamma acceptance criteria in seven out of nine patients, while the remaining two patients had gamma passing rates of at least 92%. This suggests that 4D dose calculated using uniform or sinusoidal temporal probabilities are clinically comparable to the ground truth. The reason for the lower passing rates in two of the patients may be due to a significant difference in weighting factors between uniform distribution, sinusoidal distribution, and patient‐specific distribution. Differences between 4D dose using uniform versus sinusoidal temporal probabilities were small. Compared with the uniform temporal probability distribution, sinusoidal distribution resulted in an equal or slightly better gamma passing rate in seven out of nine patients. Figure 4 shows a plot of the PTV gamma passing rates as compared to tumor motion. Standard deviation between gamma passing rates was less than 2.5%. Because the majority of gamma passing rates were very similar, no definitive correlations could be drawn between the tumor motion and the gamma passing rate from these nine subjects.

**Figure 5:**
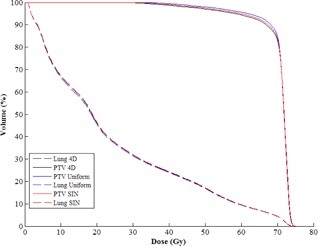
DVH comparison between dose volumes from patient specific respiratory trace (4D), uniform distribution (Uniform), and sinusoidal distribution (SIN) for subject P2.

**Figure 6:**
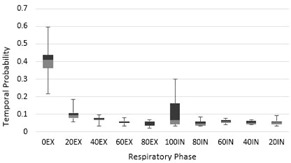
Free‐breathing patient temporal probability distributions where EX and IN refer to exhalation and inhalation phases, respectively, and preceding phase numbers refer to the percentage of full inhalation. Patients spent the majority of their time, 40.6%±10.3% on average, in the end‐exhale (0EX) phase.

When comparing to the ground truth, the absolute dosimetric differences in mean tumor dose and mean lung dose for the uniform and sinusoidal temporal probability dose distributions were within 1 Gy for all subjects, indicating that dose can be clinically approximated for the lung and the PTV using both uniform and sinusoidal temporal probability distributions. Figure 5 shows the DVH comparison between uniform, sinusoidal, and patient‐specific probability distributions, where no significant difference was found. As shown in Fig. 3, the overall difference of the sinusoidal temporal probability distribution was smaller than that for the uniform distribution. This difference may be due to patients spending more time in the end‐exhale (0EX) phase (Fig. 6), which could be better represented by the sinusoidal distribution. However, the difference between the uniform distribution and the sinusoidal distribution is small. From our results, we cannot find any correlation between the difference in dose and the magnitude of tumor motion or the size of the PTV because the differences between the uniform, sinusoidal, and ground truth dose distributions were very small. However, we recognize that the patient population in our study is limited and further investigation would be required in order to draw these conclusions.

Ideally, to compute 4D dose, the temporal probability should be derived from the patient‐specific respiratory trace. However, respiratory motion can vary from day to day, especially in patients with poor pulmonary function.^6^, ^9^ Therefore, the range of motion obtained at the time of the CT scan may vary from the range of motion observed during the time of treatment delivery. Furthermore, some institutions may find obtaining the respiratory trace an added inconvenience. Our results may suggest that 4D dose distributions can be approximated in the absence of temporal respiratory data.

In the future, it would be interesting to investigate whether the accuracy of the approximated dose distribution is in any way correlated to the tumor motion or size of the PTV. Given the limited population size of the current data, differences across the uniform, sinusoidal, and ground truth dose distributions were not sufficient to be able to draw these conclusions. By including more subjects, differences across the three 4D dose distributions may become more apparent.

## V. CONCLUSIONS

4D dose computed using uniform or sinusoidal temporal probability distribution is able to approximate 4D dose computed using the patient‐specific respiratory trace.

## ACKNOWLEDGMENTS

This work was supported by Siemens Medical Solutions USA, Inc.
